# Sudden Rupture of an Ovarian Tumor

**Published:** 1853-01

**Authors:** L. A. Dugas


*Sudden Rupture of an Ovarian Tumor—peritonitis—recovery.* By L. A. DUGAS, M. D. Mrs. D., aged about 42, the mother of thirteen children, had always enjoyed good health until the birth of her last child in March, 1851. Her delivery, although natural, was followed by considerable hemorrhage, and she has ever since felt a fixed pain or soreness in the left iliac region. At the end of a few months a distinct tumor could be perceived by pressing firmly over the painful region, and this gradually acquired a volume equal to that of a fœtal head. When turning over in bed upon the right side, a sense of dragging would always be experienced to so unpleasant a degree as to prevent her sleeping upon this side. The left lower limb would sometimes be swollen, and often feel benumbed. Sitting upon a very low seat became so uncomfortable, from the pressure of the thighs upon the abdomen, (the patient being corpulent,) that the night-glass for ordinary use was placed in a chair of usual height.

Such was the state of the patient when, on the 5th January last (1852), she was taken with uterine hemhorrhage. She was in the habit of menstruating during lactation, and had done so ever since her last confinement, with the exception of the two last periods, which induced the belief that she was now two months pregnant, and was about to miscarry. The hemhorrhage did not yield to ordinary means, but rapidly increased and became attended with uterine contractions. Ergot was now freely administered—a mole or false conception was expelled; but the loss of blood continued so excessive that fatal exhaustion appeared inevitable. A tampon was introduced and the ergot continued, which arrested the flow, but she remained pulseless, with often recurring syncope, and a cold sweat during ten or twelve hours, notwithstanding the additional free administration of brandy. The hemhorrhage was effectually stayed, and did not return upon the removal of the tampon.

The patient recovered very slowly; the anemia continued very great, and the painful annoyance in the iliac region increased. At the end of a month she was still unable to walk about the house without fatigue, and on the 4th of February was carried down stairs to a room below for a change of scene. General debility and distressing tenderness in the region of the left ovary were now the prominent features of the case. On returning to her bed-chamber in the afternoon of the 4th of February, she inadvertently sat upon a night-glass to urinate, instead of using the chair, as heretofore. As she did so, she suddenly felt a most excruciating pain throughout the entire abdomen, swooned, and fell upon the floor. On recovering, she attributed her suffering to intense cramp colic, and said she felt as if all her intestines were violently constricted or " drawn up." There was no discharge per vaginam. Enemata and warm fomentations were resorted to, and the bowels were evacuated, but without the least relief. I saw her about two hours after the accident: she had not yet been able to have her garments taken off to get in bed but was lying upon a couch. She felt a " burning, drawing pain " throughout the entire abdomen, which was exceedingly tender to the touch, but not at all tense. Her pulse was frequent, and her respiration short and thoracic. She had thrown up the contents of the stomach, but felt thirsty. The tumor could no longer be recognized by the touch. She thought she had bruised it with her thighs, in sitting upon the vessel to urinate. The fact was evident that she had not only bruised, but actually ruptured the tumor, and that its contents had escaped into the abdominal cavity, inducing peritonitis, which, in her enfeebled condition, could not be otherwise than extremely dangerous. 40 drops of laudanum were immediately administered, and a large blistering plaster applied over the abdomen ; the laudato be repeated in two hours, unless relieved.

5th Feb. The blister is well drawn; the tr. opii had to be repeated in the course of the night, and again this morning. Abdomen still very sore and somewhat full ; pulse small and very frequent. surface hot and dry; eructations and occasional vomiting ; great thirst ; breathing short and thoracic; coughing very painful. Ordered, the lateral surfaces of the abdomen, or flanks, to be covered with blistering plasters, and the anodyne to be repeated as often as necessary to mitigate the intensity of the soreness. Bi-carb. soda and lime water, alternately, in small quantities of cold water, for beverage.

6th. Local symptoms about the same, with the exception of a little increase in the volume of the abdomen. General state, better. Continue same treatment.

7th. Abdominal tenderness less marked: passed a comfortable night ; nausea relieved ; pulse not so frequent. Continue same beverage—take a little chicken broth occasionally.

12th. The peritoneal inflammation gradually subsided, but the abdomen is still tumid. No fever—patient convalescent.

May 1st. Mrs. D. is now in her usual health, but still feels a soreness in the iliac region. No tumor can now be detected. She has menstruated regularly at each period since the attack of hemorrhage, with the exception of that which came on at the time of the rupture.

It will be observed that the details of this case are given with considerable minuteness. This was necessary, in order to convey a correct idea of its nature, and to show the reader the grounds upon which the diagnosis was established. One cannot be too minute in describing cases of such rare occurrence as one in which an ovarian tumor has been ruptured by violence and emptied into the abdominal cavity, without causing death.—*Southern Medical and Surgical Journal.*

